# Multistep Chemical Processing of Crickets Leading to the Extraction of Chitosan Used for Synthesis of Polymer Drug Carriers

**DOI:** 10.3390/ma14175070

**Published:** 2021-09-04

**Authors:** Magdalena Głąb, Sonia Kudłacik-Kramarczyk, Anna Drabczyk, Martin Duarte Guigou, Agnieszka Sobczak-Kupiec, Dariusz Mierzwiński, Paweł Gajda, Janusz Walter, Bożena Tyliszczak

**Affiliations:** 1 Department of Materials Science, Faculty of Materials Engineering and Physics, Cracow University of Technology, 37 Jana Pawła II Av., 31-864 Krakow, Poland; agnieszka.sobczak-kupiec@pk.edu.pl (A.S.-K.); dariusz.mierzwinski@pk.edu.pl (D.M.); janusz.walter@pk.edu.pl (J.W.); bozena.tyliszczak@pk.edu.pl (B.T.); 2 Department of Engineering and Technology, Catholic University of Uruguay, Av. 8 de Octubre 2738, Montevideo CP 11600, Uruguay; martin.duarte@ucu.edu.uy; 3 Department of Sustainable Energy Development, Faculty of Energy and Fuels, AGH University of Science and Technology, 30 Mickiewicza Av., 30-059 Krakow, Poland; pgajda@agh.edu.pl

**Keywords:** chitosan, nisin, drug delivery systems, polymer capsules

## Abstract

Chitosan belongs to the group of biopolymers with increasing range of potential applications therefore searching for new raw materials as well as new techniques of obtaining of this polysaccharide are currently a subject of interest of many scientists. Presented manuscript describes preparation of chitosan from crickets. Obtainment of final product required a number of processes aimed at removal of undesirable substances such as waxes, mineral salts, proteins or pigments from above-mentioned insects. Chemical structure of fractions obtained after each step was compared with the structure of commercial chitosan by means of techniques such as X-ray diffraction and FT-IR spectroscopy. Final product was subsequently used for preparation of polymer capsules that were modified with active substance characterized by antibacterial and anticancer activity—nisin. Next, sorption capacity of obtained materials was evaluated as well as a release profile of active substance in different environments. Based on the conducted research it can be concluded that crickets constitute an alternative for shellfish and other conventional sources of chitosan. Furthermore, obtained capsules on the basis of such prepared chitosan can be considered as drug delivery systems which efficiency of release of active substance is bigger in alkaline environments.

## 1. Introduction

Application of materials of natural origin in wide variety of areas is one of the most interesting trends nowadays. Compounds such as polysaccharides, proteins or other bioproducts have become more and more popular. Special emphasis is directed onto chitin and its derivates [1]. One of them is chitosan which is considered as biocompatible, biodegradable and non-toxic [2,3]. These features predestine it to be a subject of increasing interest on the market of biomaterials that can be applied for biomedical purposes [4]. This deacetylated form of chitin due to its properties is useful in tissue engineering [5]. Chitosan in combination with nanomaterials forms systems that accelerate regeneration of bones [6]. Venkatesan et al. reported that chitosan-based materials can be used as carriers for delivery of growth factors during the process of bones regeneration. Mentioned systems enhanced the proliferation of cells, improving therapeutic effect [7]. Antifungal and antibacterial properties of chitosan make this biopolymer very interesting in view of its application on preparation of modern wound dressings [8,9]. Antimicrobial activity in combination with permeability to oxygen of dressings based on chitosan create an environment conducive to wound healing [10,11,12]. Introduction into chitosan-based dressings additives such as silver nanoparticles can contribute to the preparation of biomaterials exhibiting antimicrobial activity towards microorganisms such as *Staphylococcus aureus* without negative impact on human dermis cells [13].

Application of chitosan in preparation of systems of drug delivery was also well-documented [14,15]. Furthermore, growth of drug carriers in a form of capsules is observed. Sundaramurthy et al. reported about polymer capsules based on alginate as systems for encapsulation and release of drugs [16]. Moreover, Cui et al. described preparation of biocompatible and pH—responsive capsules on the basis of selected polymers and silica particles. Such prepared carriers were loaded with doxorubicin hydrochloride—well-known anticancer drug [17]. Preparation of nanoporous capsules based on polycaprolactone and L-alanine containing active substance was described by Amgoth et al. [18]. Chitosan also plays an important role in preparation of such systems. Studies on polymer capsules based on alginate and chitosan and used for delivery of low molecular molecules were presented in [19]. Synthesis of novel carriers on the basis of chitosan and poly(acrylic acid) was shown in [20].

Growing possibilities of medical application of chitosan resulted in increased demand for this polysaccharide. Therefore, new sources as well as techniques of acquiring of this biopolymer are a subject of scientists’ interest [21,22]. Interesting source of chitosan constitute naturally died honeybees that was presented in [23]. Insects were subjected to the multistep processing as a result of which Beetosan was obtained. This chitosan of bees’ origin was subsequently used for preparation of hydrogels modified with natural substances [24].

Presented publication reports preparation of chitosan from crickets. Multistage chemical processing results in obtainment material that subsequently constitutes a raw material for synthesis of polymer capsules. These can be used as carriers of active substances such as nisin. It is a peptide of natural origin that is characterized by strong antibacterial properties. Additionally, currently scientists conducted studies on its cytotoxic effect on cancer cells [25,26,27].

## 2. Materials and Methods

### 2.1. Materials

Ethyl alcohol (96%, pure p.a.), hydrochloric acid (35–38%; pure p.a.), sodium hydroxide (microgranules; pure p.a.), hydrogen peroxide (30%), citric acid (anhydrous) and calcium chloride (anhydrous, pure p.a., granules) were bought in Avantor Performance Materials Poland (Gliwice, Poland). Chitosan (low molecular weight 50,000–190,000 Da), phosphate buffer (PBS, tablets), alginate (sodium salt of alginic acid) and nisin were obtained from Sigma Aldrich S.A. (Darmstadt, Germany).

### 2.2. Preparation of Chitosan from Crickets

Chitin is one of the components that builds an external skeleton of many insects such as crickets. However, body of these animals consists of many substances including proteins, waxes and pigments therefore an extraction of chitin-chitosan complex from crickets required a multi-stage chemical process aimed at removing all undesirable substances. Here, such multistep process is presented wherein after each stage a chemical structure of the obtained material was compared to the structure of commercial chitosan using techniques such as Fourier transform infrared (FT-IR) spectroscopy via Nicolet iS5 iD7 ATR Thermo Scientific (Thermo Fisher Scientific, Waltham, MA, USA) wherein the spectra were recorded in the range of 4000–500 cm^−1^ (32 scans, resolution 4.0 cm^−1^) and X-ray diffraction. Additionally, a weight loss of the processed materials was also investigated when selecting parameters of every stage leading to the preparation of the material with the most similar structure to the structure of commercial chitosan.

#### 2.2.1. Removal of Waxes Using Soxhlet Extraction

First step of the process involved a removal of waxes by Soxhlet extraction wherein an ethyl alcohol was used as a solvent. Waxes belong to the group of hydrophobic substances and their presence in insects’ bodies can substantially hinder further reactions that are carried out using aqueous solutions. Thus, a fraction of dried crickets was introduced into the thimble and subsequently the Soxhlet extraction was conducted for different amounts of cycles. Next, obtained material was dried at room temperature and subjected to the further stages. Parameters of the process were selected considering weight losses of the processed material.

#### 2.2.2. Removal of Mineral Salts

In the second step, fraction of crickets devoid of waxes was treated with hydrochloric acid solution to remove mineral salts. Process was carried out at room temperature with constant stirring. Removal of mineral salts was performed for different periods of time and using HCl with different concentrations. Next, obtained material was washed with distilled water and dried at room temperature. Parameters of the process were selected by defining a weight loss of the processed material as well as by comparison of the structure of obtained fraction with the structure of commercial chitosan. For this purpose, X-ray diffraction and FT-IR spectroscopy were employed. 

#### 2.2.3. Removal of Proteins (Deproteinization)

In order to remove protein substances, fractions free of waxes and mineral salts were treated with sodium hydroxide. The process was conducted using 5 M NaOH solution (at elevated temperature and with constant stirring). Reaction was carried out under reflux in different periods of time. Obtained material was washed with distilled water, dried at room temperature and subjected to the XRD and FT-IR techniques.

#### 2.2.4. Removal of Natural Pigments

**Figure 1:**
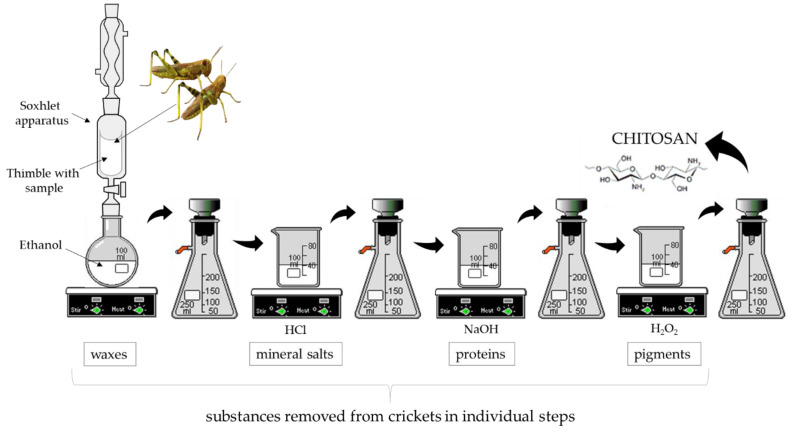
A scheme of the chemical treatment of crickets leading to the preparation of chitosan.

Final step of a chemical treatment of crickets involved a removal of natural pigments such as melanin. For this purpose, a H_2_O_2_ solution at elevated temperature was employed. Process was carried out for 1 h at elevated temperature wherein obtained material was washed using distilled water and dried at room temperature. Below, in Figure 1, a general scheme of all steps necessary for preparation of chitosan is presented.

Prepared chitosan was subsequently used for preparation of polymer capsules designed as drug carriers.

### 2.3. Determination of the Deacetylation Degree (DD) of Obtained Chitosan

The deacetylation degree (DD) of chitosan is one of the most important parameters of this biopolymer which determines its physicochemical and biological properties and thus its application potential. The value of DD corresponds to the molar percentage of monomeric glucosamine units wherein it ranges from 0 to 100 (the more deacetylated groups in the chitin structure, the higher deacetylation degree of such obtained derivative of chitin—i.e., chitosan).

In order to determine the value of DD, the acid-base potentiometric titration was performed. For this purpose, approx. 0.5 g of chitosan extracted from crickets was dissolved in 0.1 M HCl. Then, the titration using 0.1 M NaOH solution was performed with simultaneous pH measurements made using the pH-meter Elmetron CX-701 (Elmetron, Zabrze, Poland). The procedure was conducted three times.

The deacetylation degree is subsequently calculated using the following equation:

(1) $$\mathrm{DD}=161\times 10^{-3}\times \left(y-x\right)\left(\frac{C}{w}\right)\times 100$$

where

C—concentration of NaOH solution used during the titration, mol/L;
w—mass of chitosan sample, g;
x—volume of NaOH solution resulting in the first equivalent point, mL;
y—volume of NaOH solution resulting in the second equivalent point, mL;
161—molar mass of monomeric unit of chitosan which is fully deacetylated, g/mol.

Such an equation allows one to determine the number of moles of amino groups titrated in reference to the total number of moles of monomeric units (both deacetylated and acetylated ones).

### 2.4. Synthesis of Polymer Capsules Based on Chitosan Obtained from Crickets

Obtained chitosan of crickets’ origin was subsequently used for the preparation of capsules containing active substance. For this purpose, 3% solution of chitosan (mixture of commercial polysaccharide with this one extracted from insects in a weight ratio of 1:1) in 0.05% acetic acid solution, 0.5% aqueous solution of alginate, 0.1% aqueous solution of nisin and saturated solution of CaCl_2_ were prepared. Next, alginate solution and chitosan solution were mixed in suitable proportions. Then, nisin solution was added and the whole mixture was dropped slowly to the saturated solution of CaCl_2_ under continuous mixing conditions. Additionally, capsules based on alginate were also prepared and modified with nisin as reference materials for comparison with alginate/chitosan-based capsules with and without active substance. The procedure of their preparation was the same—the only difference was that the reaction mixture did not contain chitosan solution.

**Figure 2:**
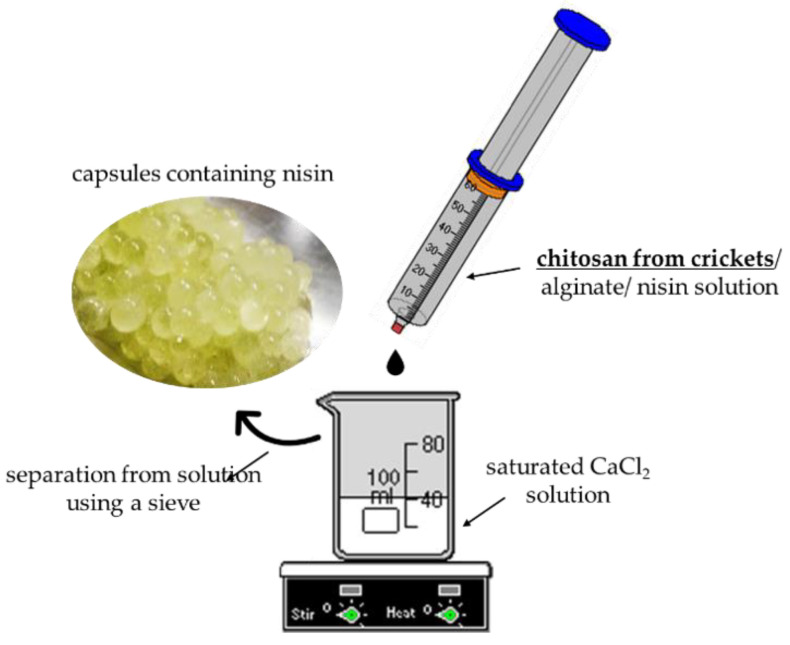
A scheme of a synthesis of polymer capsules.

In Figure 2 a scheme of a preparation of polymer capsules is presented.

Next, prepared capsules were subjected to the investigations aimed at determining their chemical structure, sorption properties and a release profile of nisin from such carriers. 

### 2.5. Studies on Polymer Capsules Based on Chitosan of Crickets’ Origin

#### 2.5.1. Characterization of a Chemical Structure of Polymer Capsules via FT-IR Spectroscopy

A presence of functional groups in the structure of prepared materials was verified using FT-IR spectroscopy. The equipment and applied conditions were the same as in the case of FT-IR analysis of fractions obtained after each step of the chemical treatment of crickets.

#### 2.5.2. Investigation on Swelling Properties of Polymer Capsules

Sorption capacity of prepared capsules is very important in viewpoint of the potential application of such materials as drug carriers. In order to characterize this property, obtained materials were immersed in distilled water. After these periods of time, swelled capsuled were placed on a moisture analyzer (apparatus Radwag MA 50.R, Radwag, Radom, Poland) wherein the drying process was conducted at 37 °C. The percentage loss of mass was determined after 15 and 30 min and corresponded to the amount of absorbed solution.

#### 2.5.3. Studies on the Release of Nisin from Capsules in Different Environments

Release profiles of nisin from the interior of chitosan/alginate-based capsules and alginate-based capsules were determined in two different environments. First environment simulated conditions occurring in human stomach—study was conducted in 2% citric acid solution (pH ~ 2); second one resembled pH which is characteristic for duodenum—such conditions were simulated by phosphate buffer (pH = 7.4). Study was carried out as follows: a suitable amount of capsules containing the highest amount of nisin solution (5 mL) were introduced into sterile vessels containing 200 mL of 2% citric acid and PBS solution and placed on a shaking incubator (Hanchen ES-60E Temperature Controlled Incubator and Shaker Scientific Incu-Shaker Shaking Incubator). Vessels were tightly closed with parafilm to limit the possible impact of the external environment. The release process was performed at 37 °C so at the temperature simulating conditions occurring in human body and at a shaking speed of 80 rpm. After every 15 min 3 mL of tested solution were taken, introduced into the cuvette and concentration of active substance in tested sample was determined using UV-Vis spectrophotometry (spectrophotometer UV-Vis Thermo Scientific Evolution 201, Thermo Fischer Scientific, Waltham, MA, USA) wherein maximum absorbance characteristic for nisin was verified at approx. λ = 230 nm. Importantly, in each case after sampling the medium, in which the release process was performed, was supplemented with 3 mL of 2% citric acid or PBS solution so that the total volume of the release medium remained constant. To sum up, the study was conducted to verify the drug release ability of both chitosan/alginate-based capsules and alginate-based capsules in various environments simulating in pH and temperature conditions occurring in human body. This, in turn, allowed to determine conditions ensuring the most effective release of the active substance.

## 3. Results and Discussion

### 3.1. Removal of Waxes from Crickets

Examples of weight losses resulting from the process aimed at removing waxes including different amount of cycles are shown in Table 1.

Weight losses calculated after extraction conducted for 4 and 5 cycles were very similar and were within the range 33.2–35.5%. Thus, it can be stated that four extraction cycles are enough for removal of waxes from processed raw material. Importantly, this is also favorable from an economical viewpoint of view and is in line with the principles of Green Chemistry because lower amount of cycles required lower energy demand. These are the reasons due to which 4 extraction cycles were chosen as a process aimed at removal of waxes from the starting material.

### 3.2. Removal of Mineral Salts

Results of the second step of processing of crickets including time, concentration of HCl solution applied, amount of extraction cycles as well as a difference between a mass of starting material and a mass of fraction obtained after demineralization are summarized in Table 2. Importantly, fraction obtained after five extraction cycles also was processed in such a way in order to check an impact of the process of removal of waxes on the course of the demineralization process.

Based on the obtained results it can be stated that weight losses of processed materials were significantly lower compared to those ones calculated after the removal of waxes. Thus, it may be concluded that crickets contained a relatively lower amount of mineral salts than waxes. Next, it can be reported that the lowest differences between mass of the starting material and the mass of the fraction after demineralization were observed in the case of processes conducted for 30 min using 2 M HCl (sample 2 and sample 6). Additionally, it was observed that the amount of cycles during the extraction had no significant impact on the course of the removal of mineral salts, so four extraction cycles were again considered as sufficient.

**Figure 3:**
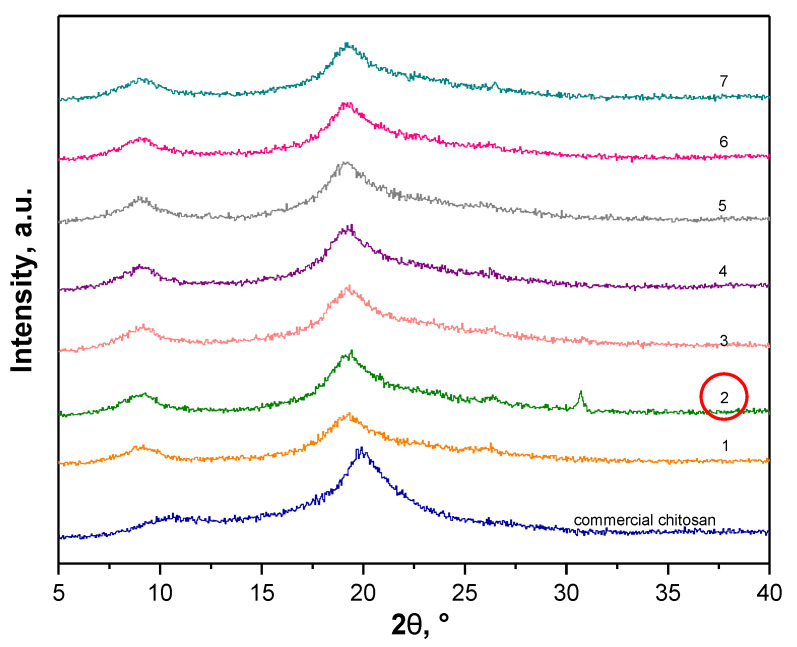
XRD analysis of obtained fractions compared to the diffraction pattern of commercial chitosan.

Nonetheless, an important aspect of performed investigations was an impact of the conditions applied on the structure of final material thus X-ray diffraction and FT-IR spectroscopy were performed to compare the chemical structure of obtained fractions with the structure of commercial polysaccharide. Obtained XRD patterns are presented in Figure 3.

Obtained diffraction patterns allowed to conclude enabled the statement that chemical structure of the fractions obtained by means of the above-mentioned parameters were very similar and relatively close to the XRD diffraction pattern of commercial chitosan. On all XRD diffractograms pattern peaks at approx. 2θ = 9° and 2θ = 19° were observed which correspond to the XRD pattern of standard chitin. Importantly, XRD peaks characteristic for chitosan are at approx. 2θ = 10° and 2θ = 20° (JCPDS # 039-1894) [28,29].

Additionally, a pattern peak at approx. 2θ = 26° (very slight) and 2θ = 31° was observed for sample 2. XRD peaks characteristic for chitin are also at approx. 2θ = 23° and 2θ = 26° [30] thus it was assumed that such peaks may also be assigned to these characteristics for chitin while their shift may be caused by the fact that the tested fraction apart from chitin consisted also of other components such as pigments or proteins which will be removed in the next steps of the performed chemical treatment. Therefore, due to the fact that the XRD pattern of sample 2 contained probably four pattern peaks characteristic for chitin and, notably, it was stated that at this step of the process the tested material should resemble chitin more than chitosan, sample 2—the sample obtained as a result of 4 extraction cycles and demineralization performed using 2 M HCl for 30 min—was selected for further process.

**Figure 4:**
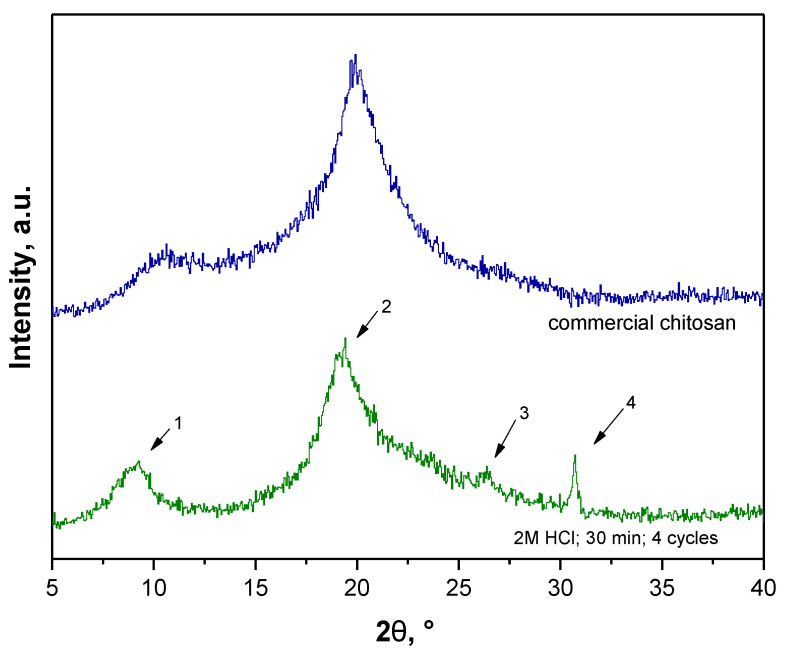
XRD diffraction patterns of commercial chitosan (blue) and selected fraction obtained using the most favorable parameters (green).

XRD diffractogram of this sample and XRD diffractogram of commercial chitosan were presented separately once again in Figure 4 for better visualization.

**Figure 5:**
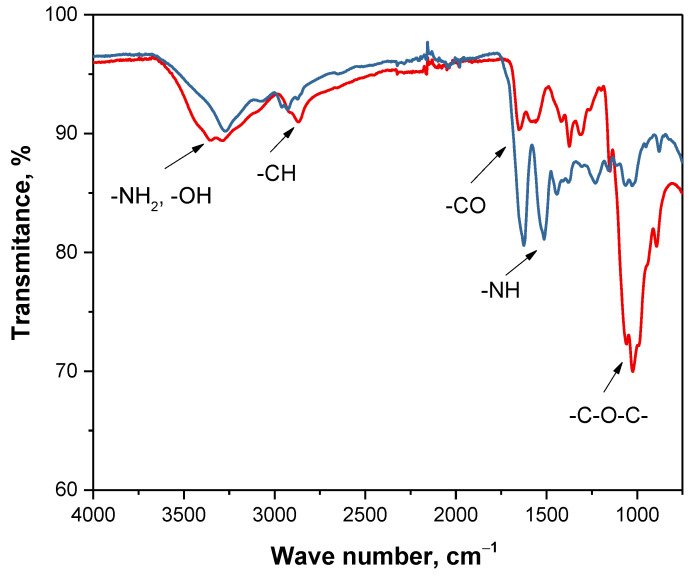
FT-IR spectra of commercial chitosan (red line) and selected sample after two-stage processing (blue line).

Next, FT-IR analysis of sample 2 was carried out. Based on the obtained spectra it was possible to determine a presence of characteristic functional groups in the structure of obtained material and compare its spectrum with spectrum of commercial compound. In Figure 5 both obtained FT-IR spectra are shown. Summary of the vibrations corresponding to the particular functional groups in the structure of tested materials is presented in Table 3.

Considering results of FT-IR analysis it can be reported that spectrum of fraction obtained after removal of waxes and mineral salts is very similar to this one deriving from commercial chitosan. On both spectra, peak characteristics for the same functional groups were observed. In both cases a broad band at 3250 cm^−1^ corresponding to the stretching vibrations of -NH_2_ and -OH groups can be observed. Moreover, bands at 1650 cm^−1^ can also be noticed for both materials which corresponds to the stretching vibrations of -CO group in the acetylamide. When comparing the intensity of these two peaks, it can be concluded that the material obtained has more acetylamide groups compared (band with higher intensity) to the commercial chitosan. It is probably caused by the fact that intensity of this band decreases with the increase of the degree of N-deacetylation of the chitin and commercial chitosan is deacetylated form of chitin so has a higher N-deacetylation degree. In turn, tested fraction at this stage of the chemical treatment resembles chitin more than chitosan which confirms results of XRD analysis. On the spectrum of commercial chitosan as well as on the spectrum of the tested sample band within the range 1100–1000 cm^−1^ derived from stretching vibrations of the -C-O-C- group in the glycosidic linkage was also observed. In the case of analyzed fraction this peak has a low intensity compared to this one of commercial polysaccharide. This may be a result of the presence of a number of organic compounds such as proteins or pigments in the tested material (they will be removed in the course of further processing), which probably cause a signal weakening.

### 3.3. Removal of Proteins

Results of deproteination of fractions deprived of waxes and mineral salts are presented below. As in the case of previous stages, a yield of conducted processes as well as a chemical structure of obtained material were considered when selecting the most favorable parameters. Table 4 shows results of removal of proteins for different periods of time.

Based on the above-presented results it may be concluded that process conducted for 20 h resulted in majority cases in higher yields. However, this may also indicate that a material was not sufficiently purified from proteins. According to the literature reports crickets are characterized by a high amount of proteins in the body therefore such a stage is a very important in viewpoint of their further processing and studies. This was a reason why it was stated that process should be carried out longer so for at least 45 h. Therefore, only fractions obtained after deproteinization performed for 45 h, 55 h and 65 h were subjected to the XRD analysis.

**Figure 6:**
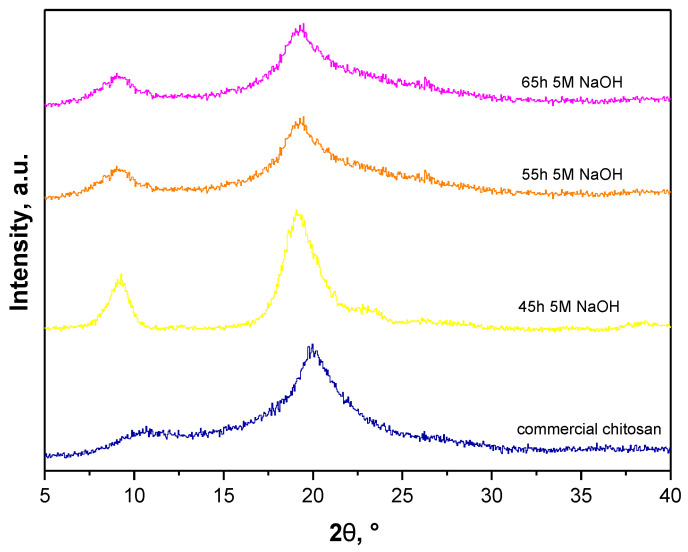
X-ray diffractograms of commercial polysaccharide (blue line) and selected samples after deproteinization process.

In Figure 6, X-ray diffractograms of the commercial chitosan and analyzed sample are shown.

As it may be noticed on all XRD diffractograms peaks at approx. 2θ = 19° and 2θ = 9° may be observed so at values corresponding to peaks characteristic for chitin. Nonetheless, on XRD patterns of samples treated with 5 M NaOH for 55 h and 65 h second peak is slightly closer to 2θ = 20°. Moreover, the first visible peak is much more like a peak at approx. 2θ = 10° visible on XRD pattern of commercial chitosan. Importantly, this peak is relatively wide for commercial chitosan. Additionally, on XRD pattern of sample treated with NaOH for 45 h a small wide peak at approx. 2θ = 23° is also visible which is characteristic for chitin—it was not observed on XRD patterns of samples treated with NaOH for 55 h and 65 h (longer time of treatment with NaOH resulted in more effective deacetylation of chitin to chitosan). Thus, the sample subjected to the deproteinization performed for 55 h and 65 h was considered for further experiments. However, when selecting the most favorable conditions of each process it is significant to consider principles of Green Chemistry. That is, when there is no difference between fraction after removal of waxes for 55 h and 65 h then the shorter time of the reaction should be selected. This, in turn, provides lower energy demand and thus is more ecological and economical. Therefore, the sample after 55 h deproteinization was subsequently analyzed using FT-IR spectroscopy.

**Figure 7:**
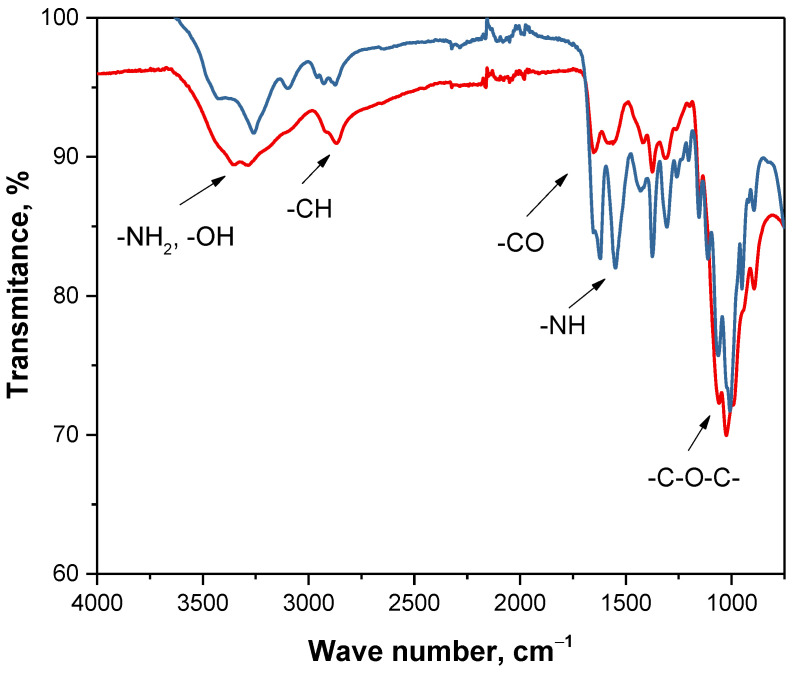
FT-IR spectra of commercial chitosan (red line) and obtained sample deprived of waxes, mineral salts and proteins (blue line).

In Figure 7 FT-IR spectra of the commercial chitosan and sample after 55 h deproteinization are presented.

It may be observed that FT-IR spectra in Figure 7 are very similar. Both on FT-IR spectrum of commercial chitosan and analyzed sample peaks corresponding to the same groups can be observed. Nonetheless, bands of analyzed sample are characterized by significantly less intensity compared to these deriving from commercial material. It is probably a result of a fact that tested material contained other substances such as pigments that were not removed.

On both spectra a wide band at 3350 cm^−1^ which can be attributed to the stretching vibrations of -OH and -NH_2_ groups was observed. Next, bands at 2870 cm^−1^ corresponding to the stretching vibrations of the methylene groups assigned to the chitosan pyranose ring were also visible. Bands at 1650 cm^−1^ corresponding to the stretching vibrations of C=O group as well as deformation vibrations of the -NH group were also observed. Additionally, bands at 1580 cm^−1^ characteristic for bending vibrations of -NH deriving from the amide group were noticeable. What is more, the band at 1347 cm^−1^ corresponded to the deformation vibration of C-H group in the methyl group of the N-acetyl group of chitosan. The broad band in the range of 1125–1025 cm^−1^ resulted from the stretching vibrations of the glycosidic linkage C-O-C. Moreover, bands occurring at 880 cm^−1^ corresponded to the stretching vibrations of C-N group.

### 3.4. Removal of Pigments

**Figure 8:**
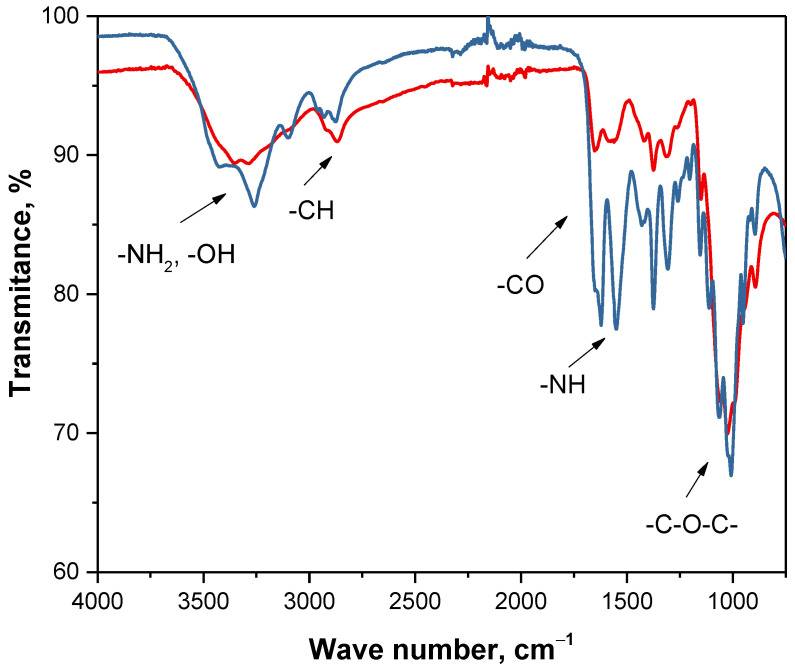
Comparison of FT-IR spectra of commercial chitosan (red) and sample after the whole processing (blue).

In the last step, the sample deprived of waxes, mineral salts and proteins selected, based on the previously performed processes—the sample after four extraction cycles, demineralization for 30 min using 2 M HCl and 55 h deproteinization—was subjected to the reaction with H_2_O_2_ solution. Reaction was carried out for 1 h at an elevated temperature while a yield was 95.17%. Obtained fraction was analyzed in the same way as previously—by means of FT-IR spectroscopy. In Figure 8 spectra of commercial chitosan and sample after removal of waxes, mineral salts, proteins and pigments are shown.

In Table 5, summary of observed vibrations with their type and corresponding functional groups is presented.

**Figure 9:**
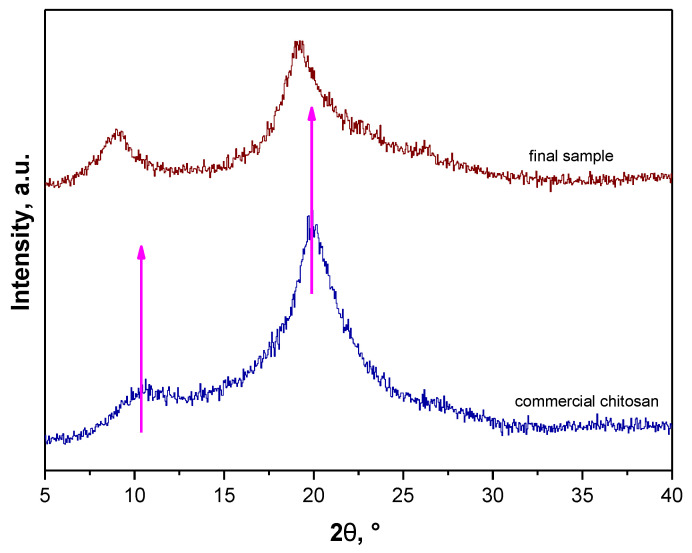
Results of XRD analysis of final sample (brown) and commercial chitosan (blue).

On both spectra vibrations, characteristics for the same groups were observed. Thus, it may be tentatively concluded that material obtained as a result of multistep processing of crickets can be defined as chitosan and, importantly, the mentioned insects can be a source of this polysaccharide. Differences in the intensity of observed peaks can be a result of a presence of slight residues of other organic substances that were not removed during the processing. Next, XRD analysis was performed for obtained final fraction. In Figure 9, diffraction patterns of commercial chitosan and sample after the whole processing are shown.

Any significant differences between obtained XRD diffraction patterns of samples before and after depigmentation were not observed. Two slightly shifted peaks in comparison to peaks visible on diffractogram of commercial chitosan were observed. Thus, it can be concluded that processed insects contained slight residues of pigments.

**Figure 10:**
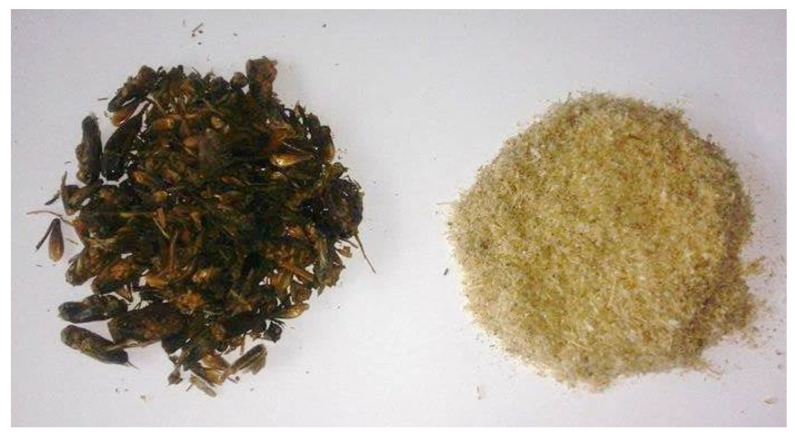
Images of crickets (**left**) and final product after multistep processing (**right**).

In Figure 10, images of crickets (raw material before processing) and final product—chitosan—are presented.

Chitosan derived from crickets was used in the next part of presented studies as a raw material for synthesis of polymer capsules that could be considered as drug carriers.

### 3.5. Determination of the Deacetylation Degree (DD)

**Figure 11:**
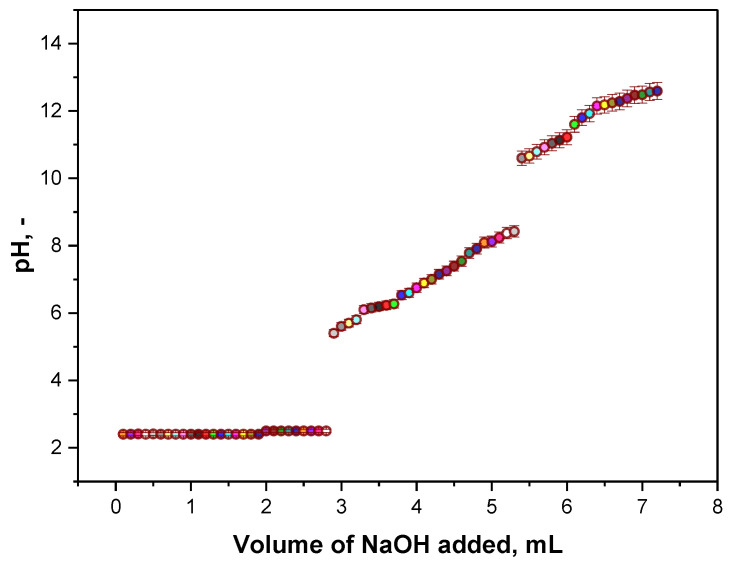
Results of the potentiometric titration of chitosan solution.

Results of the performed titration are presented in Figure 11 as a titration curve of measured pH values vs. the volume of NaOH added.

In Figure 11, the titration curve obtained as a result of performed potentiometric titration is shown. Two equivalent points can be observed on the curve. The first one corresponds probably to the neutralization of the H^+^ ions (derived from HCl) by OH^−^ ions (from NaOH) according to the following reaction:

(2) $$H^{+}+{\;\mathrm{OH}}^{-}\;\to {\;H}_{2}O$$

The second equivalent point may be assigned to the protonation of -NH_2_ groups deriving from glucosamine residues of chitosan. Firstly, -NH_2_ groups are protonated (-NH_2_ + H^+^ → -NH_3_^+^). Next, when NaOH is added, -NH_3_^+^ ions are neutralized by OH^−^ ions and therefore in such a manner the concentration of -NH_2_ groups may be determined.

Analyzing the titration curve obtained and two equivalent points visible on the curve the deacetylation degree (DD) calculated using the Equation (2) was 74.5±1.2%.

### 3.6. Synthesis of Polymer Capsules

In Table 6 compositions of prepared capsules are presented.

Prepared capsules were separated from the CaCl_2_ solution and subjected to the further studies.

### 3.7. Analysis of Chemical Structure of Polymer Capsules via FT-IR Spectroscopy

**Figure 12:**
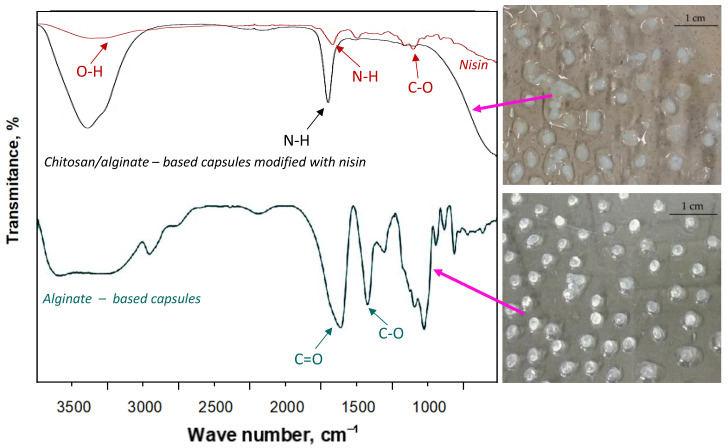
FT-IR spectra of nisin (red), chitosan/alginate-based capsules (black) and alginate-based capsules (blue) (**left**) and corresponding images of capsules (**right**).

Prepared chitosan/alginate-based capsules modified with nisin were subjected to the FT-IR analysis. Additionally—for comparison—spectroscopic analysis was also performed for alginate-based capsules without an active substance. In Figure 12, FT-IR spectra of analyzed materials are presented (left) as well as photographs showing obtained capsules (right).

Functional groups characteristic for analyzed materials were identified at specific absorption bands and showed in Figure 12. Particular attention may be directed to the wide absorption band within the range approx. 3500–3100 cm^−1^. This band in the case of chitosan/alginate-based capsules showed higher intensity compared to the same band visible on FT-IR spectrum of alginate-based capsules. This, in turn, may indicate intense interactions between these two reagents (chitosan and alginate) due to numerous hydrogen bonds and this is a reason why this band is wider for capsules consisting of two reagents compared to the alginate-based capsules [31]. Furthermore, a disappearance of asymmetric and symmetric stretching vibrations corresponding to -CO groups deriving from sodium alginate may be observed. Lack of these bands may indicate the occurrence of electrostatic interactions between dissociated carboxylic groups of sodium alginate and protonated amino groups of chitosan [31,32]. Similarly, on FT-IR spectrum of chitosan/alginate-based capsules it is difficult to notice bands corresponding to the carboxylic groups occurring in the structure of amino acids forming nisin. This is probably caused by the interactions between an introduced active substance and a structure of polymer capsules [33,34].

### 3.8. Sorption Capacity of Prepared Capsules

**Figure 13:**
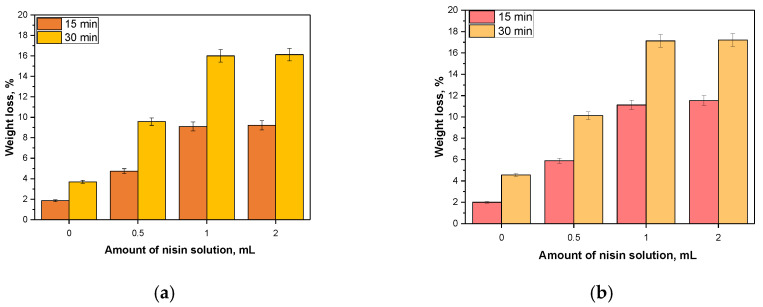
Sorption properties of prepared chitosan/alginate-based capsules (**a**) and alginate-based capsules (**b**).

Results of studies on sorption properties of capsules with special emphasis on impact of introduced additive on the tested sorption capacity is presented in Figure 13.

Obtained capsules were characterized by swelling properties. Such properties grew over time because volume of absorbed solution was higher after 30 min of swelling compared to this one observed after 15 min. Introduction of nisin into analyzed capsules resulted in an increase of their sorption capacity. It was probably caused by the fact that nisin contains in its structure many hydrophilic groups that can form hydrogen bonds with absorbed medium. Groups such as amino or carboxylic dissociate under the influence of water and resulted ions from the same groups repel electrostatically. Then, structure of the tested material becomes less compact and therefore it can absorb more water. Additionally, additive was added in the form of an aqueous solution therefore absorbed liquid can also interact with the solvent.

It may be reported that alginate-based capsules showed slightly higher swelling ability than chitosan/alginate-based capsules. Nonetheless, the differences were slight and probably due to the higher crosslinking density of capsules consisting of two components, i.e., both chitosan and alginate. It may be concluded that carboxylate groups present in the structure of alginate probably electrostatically interacted with -NH_3_^+^ groups (protonated amines) from chitosan and as a result a three-dimensional hydrogel network formed via a physical crosslinking was obtained [35]. The interactions between a cationic group from chitosan and an anionic group from alginate caused various inter- and intrachain bonds (hydrogen bonding) which, in turn, resulted in a formation of aggregates of chitosan/alginate-based capsules [36]. Considering the behavior of such structures during sorption it may be concluded that these ionic interactions between these reagents leaded to obtaining a very compact structure which sorption ability was limited. Importantly, obtained results are consistent with results presented in other works. For example, Lawrie et al. determined a swelling ability of alginate/chitosan-based capsules depending on a concentration of chitosan. It was reported that with the increase of the amount of chitosan in the composition of capsules, their swelling sorption decreased which was attributed to the ionic interactions between both biopolymers [37]. Furthermore, a structure of alginate-based capsules shows a lower crosslinking density than a structure of chitosan/alginate-based capsuled thus it may be assumed that the loosening of polymer chains is easier and as a result an absorbed liquid penetrates easily such a structure.

### 3.9. Studies on the Nisin Release from Capsules in Different Environments

**Figure 14:**
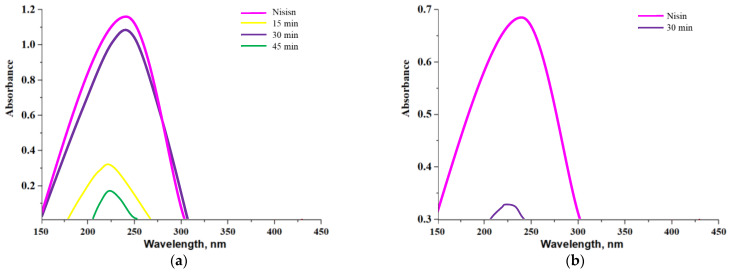
UV-Vis spectra showing a nisin release from alginate-based capsules in an alkaline environment (**a**) and in acidic environment (**b**).

**Figure 15:**
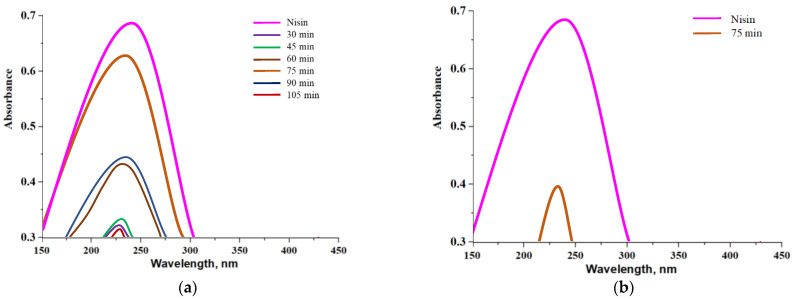
UV-Vis spectra showing a nisin release from chitosan/alginate-based capsules in an alkaline environment (**a**) and in acidic environment (**b**).

**Figure 16:**
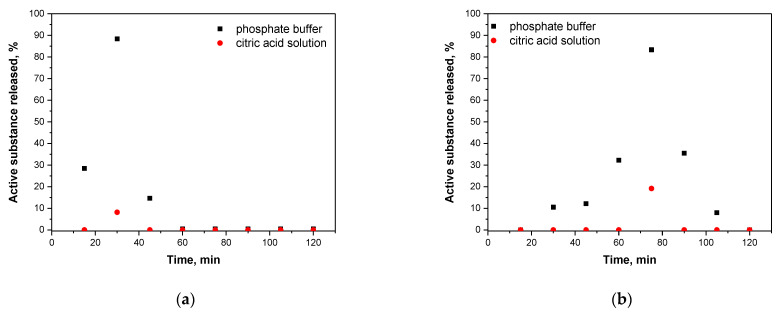
The percentage amount of the active substance released from alginate-based capsules (**a**) and chitosan/alginate-based capsules (**b**).

Results of a release of nisin from alginate-based capsules and chitosan/alginate-based capsules are presented below. In Figure 14 and Figure 15, UV-Vis spectra corresponding to the release process proceeding both in an alkaline environment (Figure 14a and Figure 15a, PBS solution) and in an acidic environment (Figure 14b and Figure 15b, 2% citric acid solution) are shown. Moreover, in order to better visualize the release process and evaluate the most beneficial conditions of such a release of active substance obtained results were also presented as graphs showing a percentage of cumulative release in time (Figure 16.)

Analyzing above-presented results it may be concluded that release of nisin is significantly more effective in slightly alkaline environment both in the case of alginate-based capsules and chitosan/alginate-based capsules. In Figure 16a a percentage release of nisin from alginate-based capsules is presented. After first 30 min of the study, the majority of the active substance was released from tested capsules in an alkaline environment while after 45 min such a release was no longer observed. In turn, in an acidic environment only 8.14% of nisin was released from analyzed carriers (in t = 30 min). Alginate, in which pKa is approx. 3.5 in such low pH, occurs mainly in a unionized form and, importantly, may precipitate as an insoluble alginic acid which, in turn, significantly limits a release of active substance [38,39]. The situation is different in an alkaline environment where alginate-based capsules show very low stability and a rapid release of big amount of nisin (i.e., 88.36%) may be observed. Moreover, such an effective release of the additive from capsules in phosphate buffer solution probably was also caused by the fact that OH^−^ ions present in tested medium enhance electrostatic repulsion occurring between COO^−^ anions (resulting from the dissociation of COOH groups occurring in the alginate structure). Therefore, a structure of capsules becomes less compact and introduced substance has more free space to get out from the polymer system can be released to the tested environment.

In the case of release process observed for chitosan/alginate-based capsules at the beginning nisin was released gradually while the release of the largest amount of this substance occurred after 75 min of the research. After 2 h of the measurements a total amount of nisin was released from tested materials. Importantly, it is worth mentioning that during the first 45 min of the research only a little substance was released and its sudden ejection was observed even after 75 min. Compared to alginate-based capsules, a maximum release of nisin from chitosan/alginate-based capsules occurs more than twice as late (is long-term) that constitutes an important information in viewpoint of the application of such systems as drug delivery systems.

Thus, it may be concluded that the application of chitosan extends the time of release of nisin from the developed systems. The pKa of the primary amino group of chitosan is approx. 6.5 and exactly this functional group affects the solubility of this polysaccharide in acidic environments. On the other hand, in neutral and alkaline environments a partial neutralization of this group as well as an aggregation of chitosan are observed that limits a release of nisin [40]. Similar results were presented by Tahtat et al. They determined a yield of insulin release both from alginate-based capsules and chitosan/alginate-based capsules. It was proved that in the case of alginate-based capsules a rapid release of an active substance in an alkaline environment was observed which was caused by a solubilization of alginate in this medium. It was also concluded that an addition of chitosan improved a stability of developed materials in alkaline solutions. This, in turn, led to the gradual release of insulin in longer time compared to such a release analyzed for alginate-based capsules (without chitosan) [41].

Due to the solubility of chitosan in an acidic environment a nisin release was possible in such medium however a percentage amount of a released active substance was only 19.16%. A slight signal deriving from the analyzed substance was noticed only after 75 min of the research. It was probably caused by the fact that in such conditions NH_2_ groups present in chitosan bind with H^+^ ions forming NH_3_^+^ cations. Such ions repel each other and as a result structure of a capsule also becomes less compact. However, the mentioned interactions are significantly less intense compared to the interactions between OH^−^ and COO^−^ that take place in an alkaline environment. It is caused by the fact that during the synthesis applied volume of alginate solution was four times bigger than volume of chitosan solution. Therefore, release of an active substance from the tested capsules was significantly better in an alkaline solution. It is important information in the viewpoint of the potential application of such materials as drug delivery systems. It may be reported that a release of a drug from such prepared materials is more effective in organs such as duodenum (pH slightly alkaline) compared e.g., to stomach (acidic conditions).

## 4. Conclusions

Crickets can be classified as a good raw material for preparation of chitosan—polysaccharide, due to its properties, is increasingly used for biomedical purposes. However, multistep chemical processing is necessary to remove from the mentioned insect substances such as waxes, mineral salts, proteins and pigments. Obtained material was characterized by properties and structure almost the same as those ones of commercial chitosan that was proved by means of the following techniques: FT-IR spectroscopy and X-ray diffraction.

Obtained chitosan of crickets’ origin was used in the next part of the research as a raw material for preparation of polymer capsules modified with nisin. Prepared capsules were characterized by good swelling properties. Introduction of nisin into the capsules resulted in an increase of sorption capacity. Based on conducted studies on release of active substance in different environments from prepared materials it can be concluded that release is significantly more effective in alkaline solutions. It is caused by the electrostatic interactions between OH^−^ and COO^−^ ions that resulted in the loosening the structure of polymer capsules. Furthermore, it was proved that a release of nisin from chitosan/alginate-based capsules occurred significantly longer than a release from alginate-based capsules (without chitosan). Importantly, a possibility of achieving an extended release profile of an active substance is undoubtedly a big advantage of the developed systems. Obtained results indicated that a maximum release of nisin from chitosan/alginate-based capsules was observed after approx. two-times longer period of time than in the case of alginate-based capsules.

Polymer capsules based on chitosan of crickets’ origin and containing nisin constitute an interesting material that can be considered for application for biomedical purposes with special emphasis on application as delivery systems of drug with antibacterial or anticancer activity.

## Figures and Tables

**Table 1:** Results of removal of waxes from crickets via Soxhlet extraction.

Process	Amount of Cycles	Weight Loss [%]
1	4	33.2
2		35.5
3		34.8
4		34.3
5		33.6
6	5	34.2
7		34.6

**Table 2:** Results of removal of mineral salts.

Sample	Time [min]	Cycles	HCl Concentration [mol/L]	Weight Loss [%]
1	60	4	1	18.5
2	30		2	13.4
3	60		2	17.9
4	30	5	1	17.7
5	60		1	20.7
6	30		2	11.7
7	60		2	20.1

**Table 3:** Vibrations of specific bonds in the analyzed materials.

Region of Absorption [cm^−1^]	Functional Group	Type of Vibration
3250–3500	-NH_2_, -OH	stretching
2900–3000	-CH	stretching
1600–1650	-CO	stretching
1500–1600	-NH	deformation
1370–1390	-CH	deformation
1300–1350	-CN	stretching
1000–1250	-C-O-C-	stretching
800–870	-CH	deformation

**Table 4:** Results of removal of proteins using 5 M NaOH solution.

Sample	Yield [%]	Time [h]	Sample	Yield [%]	Time [h]	Sample	Yield [%]	Time [h]	Sample	Yield [%]	Time [h]
1	17.18	45	6	18.11	20						
2	16.21		7	20.64		11	16.42		14	16.32	
3	16.14		8	16.91		12	16.67	55	15	16.47	65
4	17.66		9	21.32		13	16.85		16	16.17	
5	16.56		10	21.88							

**Table 5:** Summary of observed vibrations.

Wavenumber [cm^−1^]	Functional Group	Type of Vibration
3350	-OH; -NH_2_	stretching
2870	-CH_3_; -CH_2_-	stretching
1650	C=O; N-H	stretching
1580	N-H	bending
1375	C-H	deformation
1125–1025	-C-O-C-	Stretching
880	C-N	Stretching

**Table 6:** Compositions of prepared capsules.

Sample	Alginate Solution [g]	Chitosan Solution [g]	Nisin [g]
1	10.578	2.975	0
2			0.485
3			0.970
4			1.940
5	10.578	-	0
6			0.485
7			0.970
8			1.940

## Data Availability

The data presented in this study are available on request from the corresponding authors.
